# The Therapeutic Potential of *Galium verum* for Psoriasis: A Combined Phytochemical, In Silico, and Experimental Approach

**DOI:** 10.3390/ijms26157290

**Published:** 2025-07-28

**Authors:** Branislava Daskalovic, Vladimir Jakovljevic, Sergej Bolevic, Marijana Andjic, Jovana Bradic, Aleksandar Kocovic, Milos Nikolic, Nikola Nedeljkovic, Jovan Milosavljevic, Jovan Baljak, Milos Krivokapic, Svetlana Trifunovic, Jasmina Sretenovic

**Affiliations:** 1 Goodwill Pharma d.o.o., 24000 Subotica, Serbia; 2 Department of Physiology, Faculty of Medical Sciences, University of Kragujevac, Svetozara Markovica 69, 34000 Kragujevac, Serbia; 3 Center of Excellence for Redox Balance Research in Cardiovascular and Metabolic Disorders, 34000 Kragujevac, Serbia; 4 Department of Human Pathology, 1st Moscow State Medical University IM Sechenov, Trubetskaya Str. 2, 119992 Moscow, Russia; 5 Department of Pharmacy, Faculty of Medical Sciences, University of Kragujevac, Svetozara Markovica 69, 34000 Kragujevac, Serbia; 6 Department of Pharmacy, Faculty of Medicine, University of Novi Sad, Hajduk Veljkova 3, 21000 Novi Sad, Serbia; 7 Faculty of Medicine, University of Montenegro, Kruševac bb, 81000 Podgorica, Montenegro; 8 Department of Cytology, Institute for Biological Research “Sinisa Stankovic”—National Institute of Republic of Serbia, University of Belgrade, Bulevar Despota Stefana 142, 11000 Belgrade, Serbia

**Keywords:** psoriasis, *Galium verum*, in silico, morphometry, oxidative stress, rat

## Abstract

Psoriasis is a chronic inflammatory skin disorder involving oxidative stress and immune dysregulation. Given the limitations and adverse effects of conventional therapies, interest in natural treatments with anti-oxidant and immunomodulatory properties is increasing. This study aimed to comprehensively evaluate the therapeutic potential of *Galium verum* extract in an imiquimod-induced rat model of psoriasis. The extract was chemically characterized by HPLC and evaluated for anti-oxidant activity using DPPH, ABTS, and FRAP assays. Molecular docking studies targeted psoriasis-related proteins (IL-17, IL-22, IL-23, JAK2, MAPK2, NF-κB, STAT3), revealing strong binding affinities for rutin and quercetin, the extract’s dominant bioactives. In vivo, 18 *Wistar albino* male rats were divided into control (CTRL), psoriasis (PSORI), and psoriasis treated with *Galium verum* (PSORI + GV) groups. A seven-day topical application of 5% imiquimod cream was used for the induction of psoriasis. The PSORI + GV group received 250 mg/kg *Galium verum* extract orally for 7 days. Morphometric and redox analyses were performed. Histological and morphometric analyses showed reduced epidermal thickness, inflammation, and collagen content. Redox analysis revealed lowered oxidative stress biomarkers and enhanced anti-oxidant defenses. These findings suggest that *Galium verum* extract exerts anti-psoriatic effects through antioxidative and immunomodulatory mechanisms, supporting its potential as a natural adjunct therapy for psoriasis.

## 1. Introduction

Psoriasis is a chronic, immune-mediated inflammatory skin disease characterized by the hyperproliferation of keratinocytes and infiltration of immune cells, leading to the formation of erythematous, scaly plaques [1]. The etiology of psoriasis remains poorly understood [2]. However, the literature suggests that it is associated with various factors, including innate and adaptive immune responses, genetic predisposition, metabolic disturbances, and environmental influences [3]. In addition to enhanced leukocyte infiltration and elevated levels of growth factors and cytokines, several genetic elements such as IL-1β, IL-6, IL-17, IL-22, IL-23, and STAT-3 play a key role in the progression and exacerbation of psoriatic lesions [4]. Psoriasis affects approximately 2–3% of the global population. Although it is neither contagious nor life threatening, it can significantly impair quality of life and increase healthcare utilization [2].

In recent years, oxidative stress has emerged as a key factor contributing to both the pathogenesis and progression of psoriasis. It is defined as an imbalance between the production of reactive oxygen species (ROS) and the capacity of anti-oxidant systems to neutralize them. Generally, increased oxidative stress is involved in lipid peroxidation, apoptosis, tissue damage, altered T-helper cell responses, IL-17 secretion, and the degradation of cellular proteins [5]. In patients with psoriasis, numerous studies have confirmed elevated levels of oxidative biomarkers, including malondialdehyde (MDA), advanced oxidation protein products (AOPPs), and advanced glycation end-products (AGEs) [6,7]. Oxidative stress is consistently elevated in both the skin lesions and serum of psoriatic patients. These redox alterations correlate with disease severity and tend to normalize with effective treatment [5].

Standard treatment protocols for psoriasis include topical therapies for mild-to-moderate cases, phototherapy as a second-line option, systemic non-biologic agents such as methotrexate and cyclosporine for moderate-to-severe disease, and biologics targeting specific immune pathways for more refractory cases [8,9]. While these therapies have significantly improved patient outcomes, they present notable limitations, including adverse effects that limit long-term use, incomplete responses in many patients, high costs, limited accessibility of biologics, and challenges with patient adherence due to complex regimens or inconvenience [9]. These treatments are often associated with a range of side effects, including skin thinning, photosensitivity, local irritation, increased susceptibility to infections, carcinogenic potential, immunosuppression, and systemic toxicity [10].

These limitations, particularly the high cost of biologic drugs, have prompted researchers to explore alternative therapeutic strategies with a safer profile and better cost-effectiveness. One promising avenue is the use of plant-based preparations. Herbal therapies are increasingly attracting attention due to their lower toxicity, reduced side effect profiles, and capacity to modulate multiple biological pathways simultaneously [11,12]. Numerous medicinal plants are known for their anti-inflammatory, anti-oxidant, anti-angiogenic, and antimicrobial properties, supporting their potential role in the development of novel therapeutic agents. Given these advantages, ongoing research continues to focus on identifying new herbal formulations that not only alleviate disease symptoms but also improve patients’ overall quality of life [13].

The discovery of novel bioactive molecules for psoriasis treatment using in silico methods relies on the application of advanced computational techniques, which can significantly accelerate the identification and development of novel antipsoriatic agents capable of targeting multiple molecular pathways involved in the disease pathogenesis [14,15,16].

*Galium verum* (*G. verum*), also known as Lady’s bedstraw, is a perennial herb traditionally used for various medicinal purposes, including the treatment of skin disorders such as psoriasis and wound healing. Phytochemical investigations have identified a complex profile of bioactive compounds—particularly phenolic acids, flavonoids, iridoid glycosides, anthraquinones, triterpenes, luteolin, and essential oils [4,17,18,19,20]—which collectively exhibit strong anti-oxidant, anti-inflammatory, antimicrobial, and immunomodulatory activities [17,19]. These pharmacological properties support its potential use as a natural therapeutic agent for psoriasis. Notably, previous research has shown that *G. verum* extract exerts beneficial effects on both cardiovascular and dermatological manifestations of psoriasis in an experimental disease model [4].

Considering all the aforementioned findings, the aim of this study was to comprehensively assess the therapeutic potential of *G. verum* extract in psoriasis treatment through a combination of experimental and in silico methods to clarify its anti-oxidant and anti-inflammatory effects and the underlying mechanisms of action.

## 2. Results

### 2.1. Total Phenolic Content and HPLC Characterization of G. verum Extract

**Figure 1:**
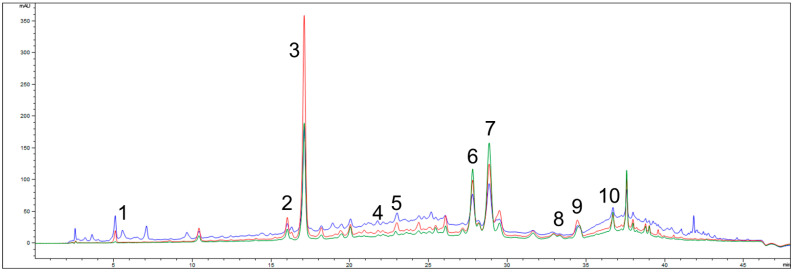
HPLC-DAD chromatogram of analyzed *G. verum* extract with detection at 280 nm (blue), 330 nm (red), and 350 nm (green); identified compounds: 1—gallic acid, 2—p-coumaric acid, 3—quercetin, 4—caffeic acid, 5—chlorogenic acid, 6—rosmarinic acid, 7—rutin, 8—quercitrin, 9—trans-cinnamic acid, 10—ferulic acid.

The TPC of *G. verum* extract was determined to be 83.21 ± 6.76 mg gallic acid equivalents (GAE) per gram of dry extract (DE). The chemical composition of the extract, analyzed by high-performance liquid chromatography (HPLC), is presented in Table 1 and Figure 1. Multiple bioactive compounds were identified, with rutin, quercetin, rosmarinic acid, and ferulic acid being the most abundant. Additionally, trans-cinnamic acid, caffeic acid, p-coumaric acid, chlorogenic acid, and quercitrin were detected at lower concentrations.

### 2.2. Anti-Oxidant Activity

The anti-oxidant activity of *G. verum* extract was evaluated using DPPH and ABTS radical scavenging assays and compared with standard anti-oxidants including ascorbic acid (AA), butylated hydroxyanisole (BHA), and Trolox. The IC_50_ values are presented in Table 2. The *G. verum* extract showed significantly higher IC_50_ values compared to AA, BHA, and Trolox in both assays (*p* < 0.05), indicating lower radical scavenging potency.

Additionally, the FRAP assay revealed a value of 1.19 ± 0.23 µM Fe^2+^ equivalents for GVE, which was lower compared to AA (2.31 ± 0.33 µM Fe^2+^ equivalents).

### 2.3. In Silico Simulations

Molecular docking calculations were performed to investigate the molecular interaction profiles of rutin and quercetin, which were identified in *G. verum* extract, with relevant biological targets involved in the pathophysiology of psoriasis. Accordingly, both focused and blind docking studies were conducted to evaluate the binding potential of the selected compounds towards interleukin receptors, JAK2, MAPK2, NF-κB, and STAT3.

#### 2.3.1. Blind Molecular Docking Studies

The molecular docking results for the most favorable binding poses of the tested compounds are presented in Table 3. A lower value for the docking score (ΔG_bind_) and a higher value for the equilibrium binding constant (K_b_) reflects a stronger molecular interaction between the compounds and selected biological targets.

In the molecular docking study, rutin exhibited the highest binding affinity toward the IL-17 receptor, as indicated by it having the lowest docking score and the highest equilibrium binding constant. This significant binding potential is further supported by the highest values of both final intermolecular energy and final total internal energy for this complex. On the other hand, the most stable ligand–protein complex was quercetin–NF-κB according to obtained docking data. A comparative analysis of the thermodynamic data for rutin and quercetin reveals that quercetin demonstrates stronger binding potential with three out of the four examined molecular (receptors for IL-22, IL-23, and NF-κB), underscoring its high affinity for these biological targets.

Rutin and quercetin complexes with the IL-17 receptor show the highest number of favorable binding contacts, with 15 and 12 interactions, respectively (Table 4). Among all non-covalent binding interactions, conventional hydrogen bonds are predominant, reflecting the polar nature of both molecules. Their hydroxyl groups, particularly within the 3,4-dihydroxyphenyl moieties and the oxychromen-4-one or chromen-4-one cores, serve as hydrogen bond donors or acceptors, forming multiple conventional hydrogen bonds. For instance, quercetin interacts with the IL-23 receptor exclusively through hydrogen bonding involving its phenolic groups. Regarding non-conventional hydrogen bonds, carbon–hydrogen, and π-donor hydrogen bonds are prevalent, as observed in the rutin–IL-17 receptor, rutin–IL-23 receptor, and quercetin–IL-22 receptor complexes (Table 3). Electrostatic interactions were observed between the carboxyl groups of Asp251 and Asp319 (NF-κB) and the aromatic rings of both rutin and quercetin (π-anion interactions), as well as between the aromatic moiety of quercetin and Asp111 of the IL-22 receptor.

**Figure 2:**
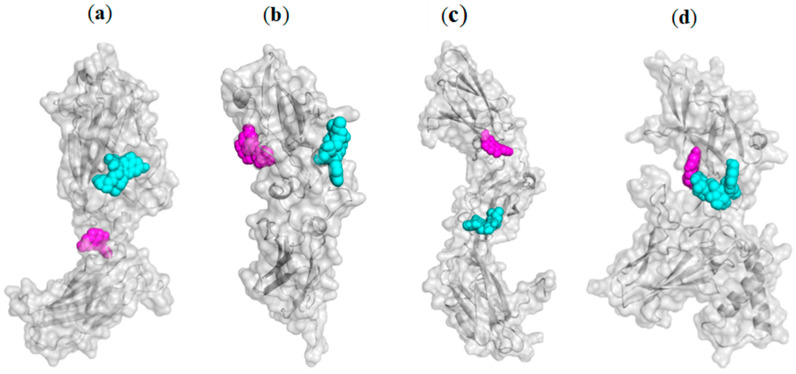
The mutual binding orientations of rutin and quercetin within the structures of IL-17 receptor (**a**), IL-22 receptor (**b**), IL-23 receptor (**c**), and NF-κB (**d**). Rutin is depicted as cyan spheres, and quercetin as magenta spheres.

Additionally, the guanidino group of Lys29 engages in a π-cation interaction with the 3,4-dihydroxyphenyl ring of quercetin. Among hydrophobic contacts, π-alkyl interactions are the most frequently observed, occurring in all complexes except for rutin–NF-κB and quercetin–IL-23 receptor. Interestingly, steric bumps were detected only in the molecular docking of quercetin into the IL-17 and IL-23 receptors, despite quercetin’s lower steric volume compared to rutin. The mutual binding orientations of rutin and quercetin within the structures of IL-17, IL-22, and IL-23 receptors, as well as NF-κB, are illustrated in Figure 2.

**Figure 3:**
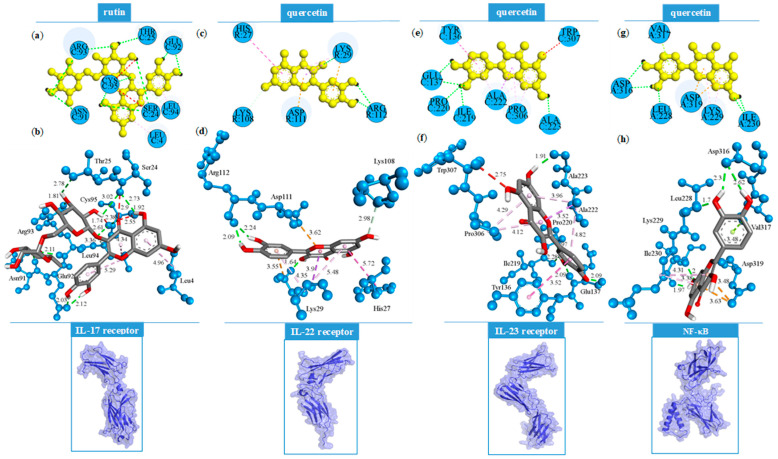
Binding interactions of the best-docked bioactive compounds with selected targets: IL-17 receptor in 2D (**a**) and 3D (**b**); IL-22 receptor in 2D (**c**) and 3D (**d**); IL-23 receptor in 2D (**e**) and 3D (**f**); and NF-κB in 2D (**g**) and 3D (**h**). The conventional hydrogen bonds (green dotted lines), electrostatic interactions (orange dotted lines), hydrophobic interactions (pink dotted lines), and steric bumps (red dotted lines) are pre-sented along with their bond lengths.

The non-covalent binding interactions of the best-docked bioactive compounds in the molecular interaction with interleukin receptors and NF-κB are presented in Figure 3.

#### 2.3.2. Focused Molecular Docking Studies

Focused molecular docking studies were conducted on three target proteins, for which crystal structures complexed with known inhibitors were retrieved from the Protein Data Bank. The investigated compounds were docked into the same binding sites as the respective co-crystallized ligands to ensure the consistent comparison of binding interactions. The calculated binding energies of the best-fitting conformations for each compound are presented in Table 5. Lower values of the docking score (ΔG_bind_) and inhibition constant (K_i_) indicate a stronger inhibition capacity of the investigated molecules toward biological targets.

Among the analyzed compounds, rutin exhibited the highest predicted inhibitory potential against the JAK2 target protein, as evidenced by the lowest docking score and inhibition constant. Additionally, the rutin–JAK2 complex demonstrated the most favorable interaction energy parameters, including the lowest final intermolecular and final total energy values among all studied complexes in the focused docking analysis. In contrast, quercetin displayed the strongest predicted binding affinity towards MAPK2, indicating a distinct target preference. Overall, rutin appears to be a more promising inhibitor of JAK2, whereas quercetin shows greater potential for inhibiting MAPK2 and STAT3.

**Figure 4:**
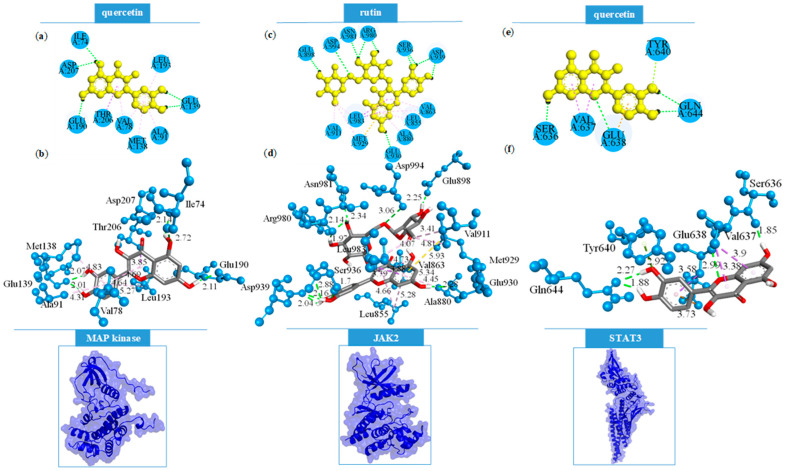
Binding interactions of the best-docked bioactive compounds with selected targets: MAPK2 in 2D (**a**) and 3D (**b**), JAK2 in 2D (**c**) and 3D (**d**), and STAT3 in 2D (**e**) and 3D (**f**). The conventional hydrogen bonds (green dotted lines), electrostatic interactions (orange dotted lines), π-sulfur interactions (yellow dotted lines), and hydrophobic interactions (pink dotted lines) are presented along with their bond lengths.

The non-covalent binding interactions established by the best-docked bioactive compounds and MAPK2, JAK2, as well as STAT3, are depicted in Figure 4.

Focused molecular docking studies identified rutin as the ligand forming the most extensive grid of interactions within the active site of the JAK2 protein, resulting in a total of 21 binding interactions (Table 6). Notably, 10 of these were conventional hydrogen bonds, primarily involving the hydroxyl groups of both the quercetin and rutinose moieties of the molecule. These hydroxyl groups predominantly acted as hydrogen bond donors, with the exception of specific interactions where the oxygen atoms of rutin functioned as acceptors, notably forming hydrogen contacts with the NH part of amide groups of the residues Ser936 and Asp994. Additional stabilizing interactions included hydrophobic contacts and a π-sulfur interaction between the π-electron-rich oxychromen-4-one core of rutin and the sulfur atom of Met929 residue. Quercetin, docked into the same JAK2 binding site, demonstrated a comparable interaction profile but with a greater emphasis on hydrophobic interactions. It formed six conventional hydrogen bonds, particularly with residues Glu930, Leu932, and Asp939, indicating a slightly less complex binding pattern compared to rutin.

Docking analyses with MAPK2 revealed that rutin and quercetin formed similarly stable complexes, with 12 and 11 total interactions, respectively, and nearly identical docking scores. Rutin established three conventional hydrogen bonds through its hydroxyl groups, acting as a donor to Glu139 and as an acceptor to Lys93 and Thr206. Furthermore, rutin exhibited a π-anion electrostatic interaction, in which the π-electrons of its oxychromen-4-one ring interacted with the carboxylate group of Glu190. Conversely, quercetin formed five conventional hydrogen bonds within the MAPK2 binding site via its phenolic hydroxyl groups. The remaining interactions were predominantly hydrophobic in nature, which may contribute to the slightly enhanced stability of the quercetin–MAPK2 complex compared to that formed with rutin.

In general, the bioactive constituents of *G. verum* exhibited the lowest binding affinity toward the STAT3 target protein in focused molecular docking analyses. Rutin and quercetin formed 9 and 10 interactions, respectively, of which 2 and 4 were conventional hydrogen bonds. Both compounds displayed highly similar binding modes. However, the slightly enhanced stability of the quercetin–STAT3 complex can be attributed to the formation of an additional hydrogen bond between the phenolic hydroxyl group of the oxychromen-4-one moiety and the carbonyl oxygen of Ser636. Furthermore, a unique π-lone pair interaction was observed between quercetin and Tyr640, a contact which is not present in any other analyzed complexes. These specific interactions likely contribute to the marginally improved binding stability of quercetin compared to rutin within the STAT3 active site.

### 2.4. Effects of the Treatment of G. verum Extract on the Skin of Psoriatic Rats

**Figure 5:**
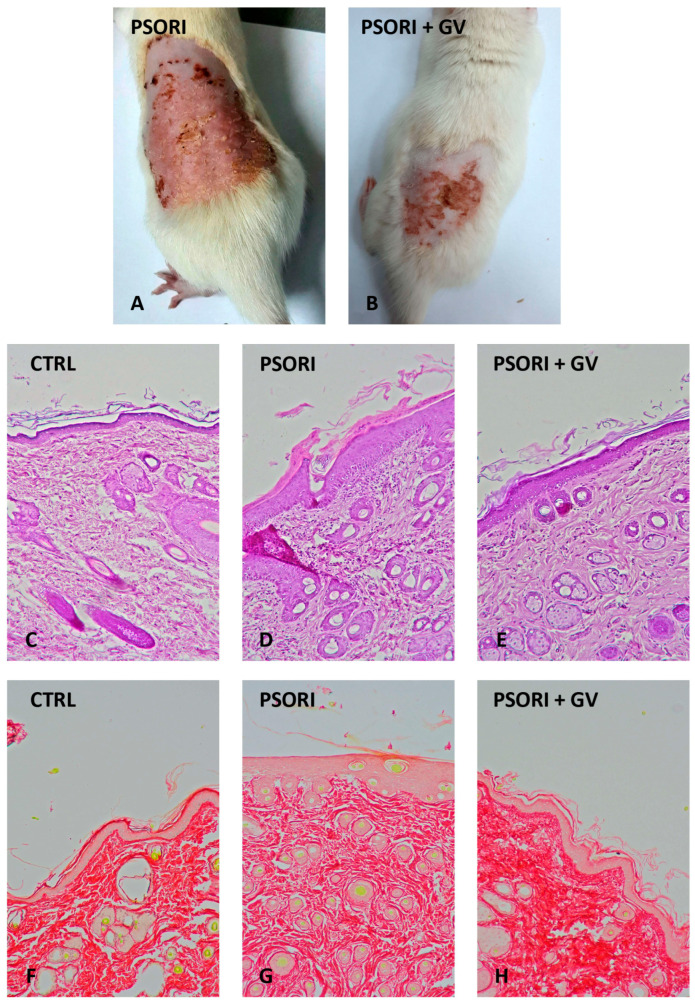
Macroscopic (**first** row) and microscopic (**second** and **third** row) images of psoriatic skin changes in rats: rat skin after psoriasis induction (**A**); rat skin after 7 days of treatment with *G. verum* extract (**B**); control group skin, H&E staining (**C**); psoriasis group skin, H&E staining (**D**); *G. verum*-treated group skin, H&E staining (**E**); control group skin, Picro Sirius Red staining (**F**); psoriasis group skin, Picro Sirius Red staining (**G**); and *G. verum*-treated group skin, Picro Sirius Red staining (**H**) (magnification 100×).

After the induction of psoriasis, the dorsal skin of rats showed marked erythema, desquamation, and increased thickness (Figure 5A and Figure S1A). After seven days of treatment with *G. verum* extract, the skin exhibited no redness or desquamation, and the thickening was reduced compared to the PSORI group (Figure 5B and Figure S1B). The PASI score following induction was nine, and after seven days of treatment, it decreased to three. Histological examination of rat skin in the control group revealed no morphological changes (Figure 5C and Figure S1C). In the PSORI group, desquamation, inflammation, and an increase in epidermal thickness (hyperkeratosis) were observed (Figure 5D and Figure S1D). In psoriatic rats treated with *G. verum* extract, reductions in epidermal thickness, inflammation, and desquamation were noted (Figure 5E and Figure S1E). On microphotographs stained with Picro Sirius Red, an increase in collagen content was observed in the PSORI group (Figure 5G), while treatment with *G. verum* extract led to a decrease in collagen content (Figure 5H), as confirmed by morphometric analysis.

### 2.5. Morphometric Analysis

**Figure 6:**
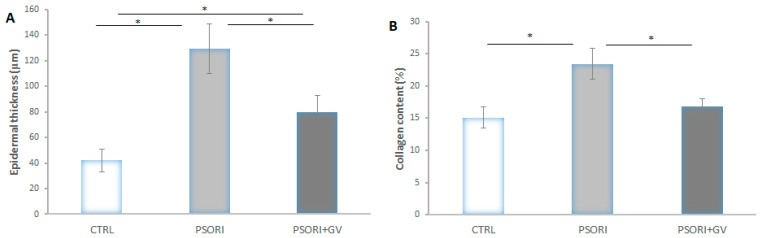
Morphometric analysis of (**A**) epidermal thickness and (**B**) collagen content. Results presented as mean value ± SD (*n* = 6). Comparation between groups was performed using a one-way ANOVA test with post hoc LSD test analysis (* denotes *p* < 0.05). Control group (CTRL), psoriasis group (PSORI), and psoriasis with *G.verum* group (PSORI + GV).

Epidermal thickness in the PSORI group increased by 207%, whereas in the PSORI + GV group it increased by 90% compared to the control values. On the other hand, a 32% decrease in epidermal thickness was observed in the PSORI + GV group compared to the PSORI group (Figure 6A). The content of collagen fibers increased by 55% in the PSORI group and by 11% in the PSORI + GV group relative to the control. A 29% reduction in collagen fiber content was observed in the PSORI + GV group compared to the PSORI group (Figure 6B).

### 2.6. Tissue Redox State Analysis

**Figure 7:**
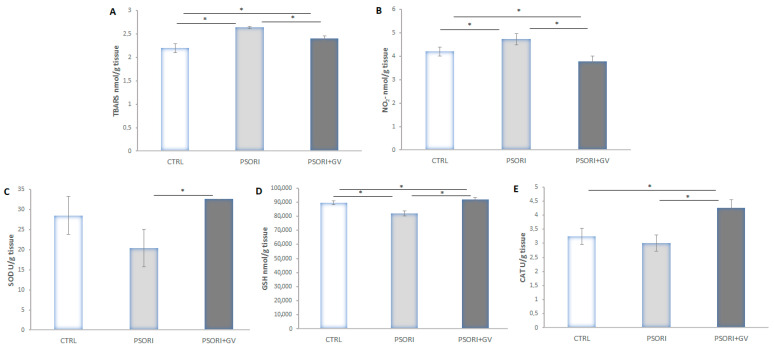
Skin tissue levels of (**A**) TBARS, (**B**) NO_2_^−^, (**C**) SOD, (**D**) GSH, and (**E**) CAT. Results presented as mean value ± SD (*n* = 6). Comparation between groups was performed using a one-way ANOVA test with post hoc LSD test analysis (* denotes *p* ˂ 0.05). Control group (CTRL), psoriasis group (PSORI), and psoriasis with *G.verum* group (PSORI + GV); (TBARS—denotes index of lipid peroxidation, NO_2_^−^—denotes nitrites, SOD—denotes superoxide dismutase, GSH—denotes reduced glutathione, and CAT—denotes catalase).

Tissue levels of TBARS (thiobarbituric acid reactive substances) (Figure 7A) and nitrites (NO_2_^−^) (Figure 7B) were increased in the PSORI group compared to the CTRL and PSORI + GV groups. Treatment with the *G. verum* extract in psoriatic rats decreased both parameters compared to the CTRL and PSORI groups. A reduction in anti-oxidant enzyme levels (catalase (CAT) (Figure 7E), superoxide dismutase (SOD) (Figure 7C), and reduced glutathione (GSH)) (Figure 7D) was observed in the PSORI group, whereas an enhancement was observed in the PSORI + GV group compared to the CTRL group.

## 3. Discussion

Herbal treatments have been used for centuries in various traditional medicine systems, including Traditional Chinese Medicine, European folk medicine, as well as Serbian folk medicine, to alleviate various skin conditions. When it comes to psoriasis, many plant-based compounds have demonstrated therapeutic potential by targeting key pathophysiology mechanisms such as oxidative stress, cytokine imbalance, and abnormal keratinocyte proliferation. In our previous study, we demonstrated the beneficial effects of chronic administration of *G. verum* extract on cardiovascular manifestations associated with psoriasis, as well as its ability to reduce inflammatory changes in the skin. In this study, we present the first comprehensive investigation of the therapeutic potential of *G. verum* extract in psoriasis by integrating in silico predictions of interactions with key psoriasis-related proteins and in vivo validation in a *Wistar* rat model. This dual approach, supported by detailed histological assessments and biochemical analyses of oxidative stress in psoriatic tissue, offers novel insights into the extract’s multi-targeted mechanisms of action.

Based on the phytochemical analysis of *G. verum* extract, which confirmed the significant presence of phenolics such as rutin, quercetin, and rosmarinic and ferulic acid, the extract was further evaluated for its in vitro anti-oxidant potential. The TPC of *G. verum* extract was determined to be 83.21 ± 6.76 mg GAE/g, indicating a moderate concentration of phenolic compounds relative to previous findings [21]. For instance, an Estonian study analyzing 50% ethanol extracts of *G. verum* blossoms reported TPC values up to 27.2 mg GAE/g, alongside strong ORAC activity (9.3 mg TE/g) [22]. In contrast, Lakic et al. reported lower TPCs (2.4–5.2 mg GAE/g) from methanolic extracts but observed strong DPPH scavenging (IC_50_ = 3.1–8.0 µg/mL), highlighting that anti-oxidant effectiveness depends not only on total phenolic content but also on the specific composition, extraction method, and solvent polarity [23].

In our study, the anti-oxidant capacity of *G. verum* extract measured by DPPH and ABTS assays revealed IC_50_ values of 87.45 ± 6.95 µg/mL and 96.21 ± 6.25 µg/mL, respectively—substantially higher (i.e., less potent) than those of standard anti-oxidants such as ascorbic acid, BHA, and Trolox. The FRAP assay further confirmed the extract’s relatively limited reducing capacity (1.19 ± 0.23 µM Fe^2+^ Eq.), in line with its moderate phenolic profile. For comparison, Turcov et al. reported a markedly higher TPC (753 mg GAE/g) in Moroccan hydroalcoholic extracts of *G. verum*, which corresponded with moderate DPPH activity (IC_50_ ≈ 59.6 µg/mL) [24]. These discrepancies across studies can be attributed to differences in plant origin, extraction solvent, plant part used, and phytochemical composition. While the in vitro anti-oxidant activity of *G. verum* extract was moderate compared to pure standards, the presence of potent bioactive compounds such as rutin and quercetin provided a basis for translating these findings into in vivo evaluation within a psoriatic rat model.

The complex pathogenesis of psoriasis remains incompletely understood, highlighting the importance of elucidating the precise molecular mechanisms underlying its development and progression. Identifying specific molecular pathways provides essential insights necessary for the development of effective, etiology-based pharmacological agents with improved therapeutic efficacy for the treatment of psoriasis. In recent years, natural compounds have attracted considerable attention in the search for novel therapies, owing to their structural diversity, favorable safety profiles, and broad availability [10]. Therefore, to explore the therapeutic potential of *G. verum* extract, a combined in silico and in vivo approach was applied to investigate thoroughly molecular interactions with psoriasis-related targets and to confirm its effects in a rat model of psoriasis.

In the first part of the current study, molecular docking studies were employed to assess the binding affinities of rutin and quercetin, as the two most abundant bioactive compounds in *G. verum* extract, toward key molecular targets involved in the etiopathogenesis of psoriasis, including interleukin receptors, JAK2, MAPK2, NF-κB, and STAT3.

It is well known that quercetin exhibits a certain therapeutic potential in the treatment of psoriasis due to its anti-inflammatory and anti-oxidant properties [25]. However, to our knowledge, the in silico analysis regarding the binding potential of quercetin for interleukin receptors, JAK2, MAPK2, NF-κB, and STAT3 has not been conducted so far. Namely, the previously published studies investigated the molecular interaction of quercetin and psoriasis genes CXCL2 and CXCR4 [26], as well as Src family tyrosine kinases (SFKs) [27]. On the other hand, Dhanabal et al. demonstrated the in vivo antipsoriatic activity of rutin using the mouse tail model of psoriasis [28]. In addition, Panhong et al. reported the molecular interaction of rutin and STAT3, suggesting its potential to downregulate the JAK2/STAT3 signaling in M5-treated HaCaT cells [29].

The blind molecular docking results demonstrate a higher binding affinity of quercetin for IL-22 and IL-23 receptors and NF-κB in comparison to rutin. On the other hand, a focused molecular docking results revealed that rutin forms the most stable complex with the JAK2 enzyme, exhibiting the lowest docking score value of −33.89 kJ/mol and the highest number of binding interactions (21 in total). Notably, rutin established key hydrogen bonds with the Asp994 residue via its phenolic groups and the amide moiety of the residue, a feature previously reported as critical for effective JAK2 inhibition. In addition to Asp994, interactions with Tyr934, Ser936, and Gly993 were also identified as relevant to complex stabilization and enzyme inhibition [30]. These findings align with previously published studies identifying rutin as a potent inhibitor of the JAK2/STAT3 signaling pathway. Notably, rutin treatment has been shown to suppress JAK2 activation, thereby reducing psoriasis-associated inflammation and abnormal keratinocyte differentiation [29].

Furthermore, both rutin and quercetin demonstrated notable affinity for MAPK2, forming comparably stable complexes with binding energies of −30.67 kJ/mol and −30.92 kJ/mol, respectively. The number of binding interactions was 12 for rutin and 11 for quercetin. Comparative analysis with a known MAPK2 inhibitor, a 2,4-diaminopyrimidine derivative, showed that effective inhibition involves hydrogen bonding with residues Leu141 and Asp142, along with additional stabilizing interactions with Lys93, Thr206, Glu104, and His108 [31]. Rutin successfully established interactions with most of these residues, including three conventional hydrogen bonds with Lys93, Glu139, and Thr206.

In the context of STAT3 enzyme inhibition, quercetin demonstrated a higher binding affinity than rutin, as indicated by its more favorable docking score (−24.39 vs. −21.92 kJ/mol). This difference may be partially attributed to the number of conventional hydrogen bonds formed—quercetin established four such interactions, while rutin formed only two. Although neither compound was able to replicate the key interactions observed in co-crystallized ligands such as SI-109 and SD-36, specifically with residues Arg609, Ser611, Ser613, and Glu612, quercetin was able to form conventional hydrogen bonds with Ser636 and Gln644. A previous study has highlighted the significance of interactions with these residues in the inhibitory activity of known STAT3 inhibitors [32]. Therefore, the ability of quercetin to engage these key residues may underlie its slightly enhanced binding affinity toward STAT3 relative to rutin.

Taken together, in silico findings suggest that rutin is likely the principal contributor to the antipsoriatic activity of the *G. verum* extract through potent inhibition of JAK2. Additionally, both rutin and quercetin appear to contribute to the extract’s pharmacological activity via MAPK2 inhibition, while quercetin showed a slightly higher affinity for STAT3. Furthermore, rutin demonstrated the most favorable interaction with the IL-17 receptor, while quercetin showed strong binding potential for IL-22 and IL-23 receptors, as well as for NF-κB. These results underscore the significant contribution of rutin and quercetin in mediating the antipsoriatic potential of *G. verum* extract.

Building on the promising in silico findings, along with detailed chemical characterization and confirmed anti-oxidant activity, the therapeutic effects of orally administered *G. verum* extract were systematically evaluated in vivo using a rat model of psoriasis, representing a logical and essential step in the preclinical assessment of its phytotherapeutic potential. Our findings indicate that systemic administration of *G. verum* extract effectively alleviated local skin alterations associated with imiquimod-induced psoriasis in rats. Throughout the treatment period, gradual improvement in clinical symptoms was observed, culminating in a notable decrease in erythema, scaling, and skin thickening by the end of the experiment. This improvement was quantitatively supported by a significant reduction in the PASI score, suggesting a clear therapeutic effect of the extract on visible psoriatic manifestations. Furthermore, histological analysis confirmed that *G. verum* extract administration led to reduced epidermal thickness and diminished inflammatory changes in the skin, providing direct evidence of its anti-inflammatory properties. These results are consistent with previously published data highlighting the anti-inflammatory potential of *G. verum* in the management of aphthous stomatitis [19] and psoriasis [4]. The therapeutic effects observed in our study are likely driven by rutin and quercetin, the major bioactive compounds in *G. verum* extract, both of which have demonstrated significant anti-psoriatic potential in previous research [25,29]. Supporting this, rutin has been shown to reduce psoriasis-associated inflammation and abnormal keratinocyte behavior, as well as to significantly improve clinical and histological outcomes by lowering PASI scores and improving histological changes [29]. Similarly, quercetin exhibits potent anti-inflammatory and anti-oxidant properties, as demonstrated in imiquimod-induced psoriasis models where it reduced PASI scores and ameliorated histopathological damage [25]. Our findings align with these studies as *G. verum* extract, rich in both rutin and quercetin, significantly improved PASI scores and histological parameters, underscoring the combined contribution of these flavonoids to the extract’s therapeutic efficacy against psoriasis.

The production of ROS plays a key role in triggering oxidative stress in psoriasis. During this process, ROS primarily function as secondary messengers, resulting in elevated levels of malondialdehyde (MDA), nitric oxide (NO), hydroxyl radicals (HO^−^), and inducible nitric oxide synthase (iNOS), while simultaneously reducing the levels of anti-oxidant defenses such as superoxide dismutase (SOD), catalase (CAT), and glutathione peroxidase (GSH-px) [33]. In our study, the levels of the lipid peroxidation index and nitrites were elevated in the group of animals with psoriasis, thus reflecting increased oxidative stress as a well-established factor contributing to the pathogenesis of psoriasis. This finding is consistent with previously published data [34,35,36]. On the other hand, treatment with *G. verum* extract significantly reduced TBARS and nitrite levels in the skin of psoriatic rats. Unfortunately, there are no available data in the current literature specifically addressing the effects of *G. verum* extract on these two pro-oxidant markers in the skin of rats with psoriasis. Vuletic et al. reported that administration of *G. verum* extract decreased the levels of TBARS in the tissue affected by aphthous stomatitis [19], which is in accordance with our result as both conditions share oxidative stress-driven inflammation. Consistent with our findings, it has been previously confirmed that quercetin effectively increased the values of GSH, CAT, and SOD, while decreasing the accumulation of pro-oxidants in the skin tissue of imiquimod-induced psoriatic mice. Therefore, it may be assumed that quercetin plays an important role in mediating the extract’s antioxidative effects [25].

Moreover, the skin acts as a protective shield and is regularly subjected to environmental stressors, making it a key location for free-radical formation. In limited concentrations, these radicals play an essential role in immune defense and aid in cell differentiation. CAT, SOD, and GSH-Px represent key anti-oxidants in the skin. GSH plays an important part in shielding the skin from oxidative damage [37]. In our study, tissue levels of GSH, SOD, and CAT were decreased in the PSORI group, confirming an impaired anti-oxidant defense system which exacerbates the inflammatory cascade and disrupts normal skin homeostasis, as previously reported [34]. Conversely, treatment of psoriatic rats with the *G. verum* extract significantly increased tissue GSH, SOD, and CAT levels, providing direct evidence of the plant’s anti-oxidant effect, which corresponds with the observed improvement in clinical psoriasis severity as indicated by decreased PASI scores. A similar effect of *G. verum* extract was confirmed in the study by Vuletić et al., which demonstrated that administration of *G. verum* extract increased the local levels of SOD, GSH, and CAT, confirming its broader potential to modulate oxidative damage [19].

Elevated levels of oxidative stress activate Th1 and Th17 cells, as well as keratinocytes, through various mechanisms, including the MAPK, NF-κB, and JAK-STAT signaling pathways. The activation of these cells triggers the production of a range of cytokines. Activation of Th1 cells stimulates the production of IFN-γ, IL-2, TNF-α, and TNF-β; activation of Th17 cells promotes the production of IL-17, IL-22, IL-23, and TNF-α; while keratinocyte activation induces the production of polymorphonuclear cells (PMNs), VEGF, IL-6, IL-8, TNF-α, and antimicrobial peptides. These cytokines further activate T-cells and mast cells, thereby promoting excessive keratinocyte proliferation, increased neutrophil formation, and chronic skin inflammation [33]. In our study, epidermal hyperplasia (hyperkeratosis), inflammation, and increased collagen content were observed in the psoriasis group, which is in accordance with the results of previous studies. It is particularly important to emphasize that increased oxidative stress induces disturbances in lipid peroxidation, which activates cGMP while simultaneously reducing cAMP levels, thereby contributing to epidermal hyperplasia in psoriasis [38]. Given that our study confirmed elevated TBARS levels, we assume that the observed epidermal hyperplasia is a consequence of oxidative stress dysregulation in the skin of rats with psoriasis. In particular, it should be emphasized that increased oxidative stress induces lipid peroxidation disorder that activates cGMP with a simultaneous decrease in cAMP, inducing epidermal hyperplasia in psoriasis. Given that an increased level of TBARS was confirmed in our study, we believe that epidermal hyperplasia is a consequence of disturbed oxidative stress in the skin of rats with psoriasis.

On the other hand, in the group of animals that were subjected to the simultaneous application of *G. verum* extract and the induction of psoriasis, it significantly reduced inflammation, epidermal thickness, and the content of collagen fibers in the skin of rats. We assume that this result is a consequence of the anti-oxidant and anti-inflammatory ability of this plant, which was confirmed in this study and also in our previous study [4]. Based on the in silico results obtained in this study, we believe that this plant exerts its effects through all three of the aforementioned mechanisms, MAPK, NF-κB, and JAK2-STAT3 signaling pathways, by reducing the production of proinflammatory cytokines generated by keratinocytes, Th17 cells, mast cells, and T-cells. These effects are likely mediated by the presence of rutin and quercetin. These represent only some of the potential mechanisms through which this plant may exert its beneficial effects in the treatment of psoriasis.

## 4. Materials and Methods

### 4.1. Extract Preparation and Characterization

For the purposes of this research, dried plant (*G. verum* L.) was purchased, pulverized, and stored in well-sealed paper bags at room temperature until the extract preparation. The ethanol extract of the abovementioned plant was obtained under the reflux of the solvent. This method involves extraction at the boiling point of the solvent (70% ethanol). The extraction was performed for 2.5 h, after which the mixture was filtered through a gauze and left at room temperature to spontaneously precipitate ballast substances. Finally, the obtained liquid extract was filtered (Whatman, No. 1, Cytiva, Maidstone, Kent, UK), while a rotary vacuum evaporator (40 °C, 90 rpm, and 250 mbar vacuum) was applied to obtain a dry extract, which was afterward stored in dark glass vials at +4 °C until further testing [17,19].

#### 4.1.1. HPLC Analysis of *G. verum* Extract

The qualitative and quantitative profiling of bioactive compounds in the obtained extracts was performed using HPLC on an Agilent HP 1100 chromatographic system equipped with a diode array detector (Agilent Technologies, Waldbronn, Germany). The analysis was based on a previously published method [39], focusing on major phenolic acids, gallic, caffeic, trans-cinnamic, p-coumaric, chlorogenic, rosmarinic, and ferulic—as well as flavonoids including quercetin, rutin, and quercitrin. The chromatographic separation was achieved using a Nucleosil C18 reversed-phase column (250 mm × 4.6 mm, 5 µm particle size). The mobile phase comprised solvent A (water with 0.1% formic acid *v*/*v*) and solvent B (methanol). Gradient elution was performed under the following program: 10% B at 0 min, linearly increased to 25% at 10 min, 45% at 20 min, 70% at 35 min, and 100% at 40 min, followed by re-equilibration to 10% B at 46 min. The flow rate was varied as follows during the run to optimize resolution: 1.0 mL/min for 0–10 min, 0.8 mL/min for 10–20 min, 0.7 mL/min for 20–30 min, and then restored to 1.0 mL/min until the end of the analysis. The injection volume was set at 5 µL. Detection wavelengths were selected based on the following specific UV absorption of each compound group: gallic, caffeic, and trans-cinnamic acids were monitored at 280 nm; p-coumaric, chlorogenic, rosmarinic, ferulic acids, and quercetin at 330 nm; and rutin and quercitrin at 350 nm. Compound identification and quantification were based on external calibration curves prepared using analytical standards analyzed under identical chromatographic conditions

#### 4.1.2. Total Phenolic Content

The TPC was determined using a microplate-adapted version of the Folin–Ciocalteu method, as previously described by Bobo-García et al. [40]. A calibration curve was constructed using gallic acid standards. For each test well in a 96-well microplate, 20 μL of the sample or standard was pipetted, followed by 80 μL of distilled water. Then, 20 μL of diluted Folin–Ciocalteu reagent was added. After a brief incubation, 80 μL of 7.5% sodium carbonate was introduced to initiate the colorimetric reaction. The plate was left at room temperature for 30 min for full color development, and absorbance was read at 765 nm. The TPC values were calculated based on the gallic acid calibration curve and expressed as milligrams of gallic acid equivalents per gram of dry extract (mg GAE/g).

#### 4.1.3. Antioxidative Activity

##### DPPH Radical Scavenging Assay

The ability of samples to scavenge DPPH radicals was assessed using a miniaturized, high-throughput microplate assay. A 0.004% (*w*/*v*) methanolic DPPH solution was mixed with test samples or reference standards in 96-well plates to a final volume of 200 μL per well. The reaction was carried out in the dark at ambient temperature for 30 min. Absorbance was then recorded at 515 nm. Anti-oxidant activity was calculated as a percentage inhibition relative to a negative control (DPPH without anti-oxidant), and the IC50 value (the concentration needed to inhibit 50% of DPPH radicals) was derived for each sample. The assay’s reliability was confirmed by comparing results with known anti-oxidant standards [41].

##### ABTS Radical Cation Decolorization Assay

The ABTS assay was used to quantify anti-oxidant capacity based on the quenching of ABTS radical cations. ABTS radicals (ABTS^+^) were generated by reacting the ABTS stock solution with potassium persulfate and incubating the mixture in the dark for 16 h. The resulting radical solution was diluted in ethanol until an initial absorbance of 0.700 ± 0.020 at 734 nm was reached. Then, 20 μL of each sample or reference standard was added to 180 μL of the ABTS^+^ solution in a 96-well plate. Absorbance was measured at 734 nm within the first minute of mixing. All measurements were performed in triplicate for statistical reliability [41,42,43].

##### FRAP Assay

The FRAP assay was employed to evaluate the reducing power of the extracts. The FRAP reagent was freshly prepared for each experiment by mixing 300 mM acetate buffer (pH 3.6), 10 mM TPTZ in 40 mM HCl, and 20 mM ferric chloride in a 10:1:1 ratio. In each well of a 96-well microplate, 10 μL of the test sample was combined with 300 μL of the FRAP working solution. The reaction was incubated at 37 °C for 10 min, after which the absorbance was measured at 593 nm. Distilled water served as the blank. Trolox was used as the positive control, and ferrous sulfate was used to establish the standard curve. Results were reported as micromoles of Fe^2+^ equivalents (µmol Fe^2+^ eq.), following the methodology described by Bolanos de la Torre et al. [44].

### 4.2. Molecular Docking Protocol

The semi-flexible docking protocol was executed using Lamarckian Genetic Algorithm with default settings in AutoDock 4.2 software [45]. Rutin and quercetin were selected for in silico calculations based on the HPLC-DAD analysis of *G. verum* extract. The 3D conformer coordinates of investigated molecules were obtained from the PubChem database (https://pubchem.ncbi.nlm.nih.gov/, accessed on 19 June 2025) in sdf file format and converted into pdb format using PyMOL 2.5.5 software [46]. Molecular docking studies were utilized to investigate the interaction of the two most abundant bioactive compounds present in *G. verum* extracts and key molecular targets involved in the pathophysiological mechanisms of psoriasis. These molecular targets include the receptors for IL-17, IL-22, and IL-23, as well as the critical protein components, Janus kinase 2 (JAK2), MAP kinase-activated protein kinase 2 (MAPK2), nuclear factor NF-kappa (NF-κB), and signal transducer and activator of transcription 3 (STAT3), which are implicated in the molecular pathways of psoriasis aetiopathogenesis [33,47,48]. The crystallographic structures of receptors for IL-17 (PDB ID: 5HI5) [49], IL-22 (PDB ID: 3DLQ) [50], IL-23 (PDB ID: 5MZV) [51], JAK2 (PDB ID: 4BBE) [30], MAPK2 (PDB ID: 3KC3) [31], NF-κB (PDB ID: 1A3Q) [52], and STAT3 (PDB ID: 6NJS) [32], were downloaded from the RCSB Protein Data Bank database (http://www.rcsb.org/, accessed on 10 June 2025). Biological data were processed using Discovery Studio [53] and AutoDock Tools [45] by removing non-essential protein chains and co-crystallized ligands. Polar hydrogen atoms and Kollman charges were subsequently added to prepare the structures for docking. Blind molecular docking studies were carried out on receptors for IL-17, IL-22, and IL-23, as well as NF-κB, using a full-sized grid box with dimensions of 126 × 126 × 126 points and a grid spacing of 0.375 Å. On the other hand, focused molecular docking studies were performed on JAK2, MAPK2, and STAT3 using a grid spacing of 0.375 Å with grid box dimensions of 40 × 40 × 40 points. The binding sites on JAK2, MAPK2, and STAT3 were defined according to the coordinates of co-crystallized ligands (PDB entries 3O4, MK2, and KQV, respectively). The grid box center coordinates for x, y, and z were defined as 3.581, −11.802, and −1.188 for JAK2, −6.235, 56.03, and −10.98 for MAPK2, and 13.498, 54.118, and 0.1 for STAT3. The best-docked binding poses of rutin and quercetin were generated using Discovery Studio to analyze non-covalent binding interactions. The binding affinity assessment was evaluated based on the category, type, and total number of non-covalent interactions, docking score (kJ/mol), equilibrium binding constant (K_b_), and inhibition constant (K_i_). The AutoDock software calculates docking score based on the following equation: ΔG_bind_ = ΔG_vdw+hbond+desolv_ + ΔG_elec_ + ΔGt_otal_ + ΔGt_or_ − ΔG_unb_, where ΔG_bind_ represents the free binding energy; ΔG_vdw+hbond+desolv_ is the sum of the energies of dispersion and repulsion (ΔG_vdw_), hydrogen bonding energy (ΔG_hbond_), and desolvation energy (ΔG_desolv_); ΔG_elec_ denotes the electrostatic interaction energy; ΔG_total_ corresponds to the final total internal energy; ΔG_tor_ represents torsional free energy; and ΔG_unb_ is unbound energy of the system. The values of K_b_ and K_i_ were calculated from docking score values using the equations ∆G = −RTlnK_b_ and ∆G = RTlnK_i_, respectively. The docking simulations were performed at 298 K, with R representing the universal gas constant with a value of 1.9872036 × 10^−3^ kcal K^−1^ mol^−1^.

### 4.3. In Vivo Experiment

#### 4.3.1. Induction of Psoriasis and Treatment

For seven consecutive days, the rats from the psoriasis groups were treated daily by topically applying 50 mg 5% imiquimod cream (Aldara) to their shaved back skin [4]. The rats from the PSORI + GV group received *G verum* extract at a dose of 250 mg/kg body weight [54] daily by oral gavage for seven days 4 h before the application of imiquimod [38]. The selected dose of the extract was based on a previously published study reporting confirmed anti-oxidant activity [54]. Psoriasis area severity index (PASI score) was calculated at the end of experiment. The following parameters were monitored, erythema, scaling, and thickness of back skin, and they were scored on a scale from 0 to 4 for each parameter (0—none; 1—slight; 2—moderate; 3—marked; and 4—very marked), so the maximum PASI score was 12 [4].

#### 4.3.2. Tissue Redox State

Skin samples from all animals were stored at −80 °C until analysis. The tissue was homogenized in phosphate-buffered saline (PBS, pH 7.4; 1:10 *w*/*v*) on ice using an electric homogenizer. The homogenates were then centrifuged at 1200× *g* for 20 min at 4 °C. The resulting supernatant was collected for analysis (using a Shimadzu UV-1800 spectrophotometer, Shimadzu, Kjota, Japan) to assess tissue levels of lipid peroxidation (TBARS), nitrites (NO_2_^−^), and the activities of catalase (CAT), superoxide dismutase (SOD), and reduced glutathione (GSH) [55].

##### Lipid Peroxidation Index (TBARS)

Lipid peroxidation levels in tissue homogenates were assessed by measuring TBARS using 1% thiobarbituric acid (TBA) dissolved in 0.05 M NaOH. To prepare the TBA extract, 0.4 mL of the sample was mixed with 0.2 mL of trichloroacetic acid, followed by a 10 min incubation on ice. The mixture was then centrifuged at 6000 rpm for 15 min. The resulting supernatant was incubated at 100 °C for 15 min, and absorbance was measured at 530 nm. Distilled water was used as a blank control [55,56].

##### Nitrite (NO_2_^−^) Determination

Nitrite levels, serving as an indicator of nitric oxide production, were quantified using the Griess reagent. For analysis, 100 µL of 3 N perchloric acid (PCA), 400 µL of 20 mM ethylenediaminetetraacetic acid (EDTA), and 200 µL of tissue homogenate were combined and placed on ice for 15 min. The mixture was then centrifuged at 6000 rpm for 15 min. After discarding the supernatant, 220 µL of potassium carbonate (K_2_CO_3_) was added. Absorbance was measured at 550 nm, using distilled water as a blank [55,57].

##### CAT Activity

CAT activity was assessed following the method described by Aebi [55]. A diluted tissue homogenate (1:7 *v*/*v*) was treated with a chloroform–ethanol mixture (0.6:1 *v*/*v*). The reaction mixture consisted of 50 µL of CAT buffer, 100 µL of the homogenate, and 1 mL of 10 mM hydrogen peroxide (H_2_O_2_). Absorbance was measured at 360 nm, and catalase activity was expressed in units per gram of tissue (U/g tissue) [55].

##### SOD Activity

SOD activity was determined using the epinephrine autoxidation method as described by Beutler [55]. A 50 µL sample of testicular homogenate was mixed with 1 mL of carbonate buffer, followed by the addition of epinephrine. The absorbance was read at 470 nm, and the enzyme activity was expressed as U/g tissue [55].

##### GSH Level

The level of GSH was measured using the method based on its oxidation by 5,5′-dithiobis-(2-nitrobenzoic acid) (DTNB), according to Beutler [34]. For GSH extraction, 0.1 mL of 0.1% EDTA, 400 µL of homogenate, and 750 µL of precipitation solution (containing 1.67 g metaphosphoric acid, 0.2 g EDTA, and 30 g NaCl, dissolved in distilled water up to 100 mL) were combined. The mixture was vortexed, kept on ice for 15 min, and then centrifuged at 4000 rpm for 10 min. Distilled water was used as the blank. The absorbance was measured at 420 nm, and GSH levels were expressed accordingly [55].

#### 4.3.3. Histology and Morphometry

Skin samples were fixed in 4% neutral-buffered formalin, then dehydrated through a graded ethanol series (70%, 96%, and 100%), cleared in xylene, and embedded in Histowax^®^ (Histolab Product AB, Göteborg, Sweden) for subsequent histological examination. Sections of 5 µm thickness were stained with Hematoxylin and Eosin (H&E) to assess morphological alterations. To evaluate collagen content, Picro-Sirius Red staining was employed, producing red-stained collagen fibers, dark brown to black nuclei, and yellowish cytoplasm. The staining protocol included deparaffinization and rehydration of tissue sections using ethanol (100%, 96%, and 70%). Following rehydration, sections were incubated in Weigert’s hematoxylin for 5 min, then rinsed in distilled water for 10 min. Subsequently, samples were stained with Picro-Sirius Red for 60 min, rinsed again in distilled water for 10 min, and treated twice with 2% glacial acetic acid (5 min each). Final steps included washing in distilled water, dehydration in ethanol, clearing in xylene, and mounting with DPX. Tissue images were captured using a digital camera mounted on an Olympus BX51 microscope, and morphometric analysis was performed using Image Pro-Plus software 7.0 (Media Cybernetics, Rockville, MD, USA).

## 5. Conclusions

This study demonstrates that *G. verum* extract possesses significant therapeutic potential in the treatment of psoriasis, as evidenced by its anti-oxidant and anti-inflammatory effects. The extract improved clinical and histological parameters of diseases, including reductions in erythema, scaling, epidermal thickness, and inflammation, whereas also normalizing oxidative stress biomarkers. In silico, analysis further supported these findings, suggesting that the observed effects may be mediated through inhibition of the MAPK, NF-κB, and JAK2-STAT3 signaling pathways, primarily via the bioactive compounds rutin and quercetin. Taken together, these results highlight the potential of *G. verum* as a promising candidate for the development of phytotherapeutic strategies against psoriasis. However, further investigations are needed to confirm these findings.

## Figures and Tables

**Table 1:** Quantitative and qualitative evaluation of the components found in *G. verum* extract, expressed as mg/g of dry extract.

Name of Compound	*G. verum* Extract
Rutin	18.85 ± 1.51
Quercetin	6.60 ± 0.46
Rosmarinic acid	4.80 ± 0.29
Ferulic acid	3.43 ± 0.21
Gallic acid	1.03 ± 0.15
Trans-cinnamic acid	0.41 ± 0.04
Quercitrin	0.35 ± 0.02
p-coumaric acid	0.21 ± 0.02
Chlorogenic acid	0.09 ± 0.0
Caffeic acid	0.06 ± 0.0

**Table 2:** Anti-oxidant activity of *G. verum* extract and standards.

Investigated Samples and Standards	DPPH IC_50_ (µg/mL)	ABTS IC_50_ (µg/mL)
GVE	87.45 ± 6.95 ^a,b,c^	96.21 ± 6.25 ^a,b,c^
AA	9.15 ± 0.72	10.19 ± 0.99
BHA	11.29 ± 1.03	12.46 ± 1.07
Trolox	5.33 ± 0.18	7.36 ± 0.84

GVE—*G. verum* extract, AA—ascorbic acid; BHA—butylated hydroxyanisole. Data are presented as Mean ± SD. Normalities were confirmed using the Shapiro–Wilk test, prior to an ANOVA with Tukey post hoc test. ^a^—significant difference at the level *p* < 0.05 in comparison with AA; ^b^—significant difference at the level *p* < 0.05 in comparison with BHA; ^c^—significant difference at the level *p* < 0.05 in comparison with Trolox.

**Table 3:** Blind molecular docking parameters for the best-docked binding poses of *G. verum* bioactive compounds.

Ligand–Protein Complex	ΔG_bind_ (kJ/mol)	K_b_ (M^−1^)	ΔG_Intermol. Energy_ _(vdw+Hbond+desolv)_ (kJ/mol)	ΔG_elec_ (kJ/mol)	ΔG_Final Intermol. Energy_ (kJ/mol)	ΔG_total_ (kJ/mol)	ΔG_tor_ (kJ/mol)	ΔG_unb_ (kJ/mol)
Rutin–IL-17 receptor	−25.78	3.29 × 10^4^	−41.84	−2.30	−44.14	−27.32	18.36	−27.32
Rutin–IL-22 receptor	−17.94	1.39 × 10^3^	−34.43	−1.88	−36.31	−25.31	18.37	−25.31
Rutin–IL-23 receptor	−15.90	6.10 × 10^2^	−34.06	−0.21	−34.27	−28.41	18.37	−28.41
Rutin–NF-κB	−22.85	1.00 × 10^4^	−39.25	−1.97	−41.22	−22.68	18.37	−22.68
Quercetin–IL-17 receptor	−23.35	1.24 × 10^4^	−29.20	−1.05	−30.25	−9.16	6.90	−9.16
Quercetin–IL-22 receptor	−24.73	1.37 × 10^3^	−30.12	1.51	−31.63	−9.07	6.90	−9.07
Quercetin–IL-23 receptor	−23.73	1.44 × 10^4^	−30.21	−0.42	−30.63	−7.78	6.90	−7.78
Quercetin–NF-κB	−25.86	3.39 × 10^4^	−30.88	−1.88	−32.76	−9.37	6.90	−9.37

**Table 4:** Overview of non-covalent binding interactions established by *G. verum* bioactive compounds and receptors for IL-17, IL-22, IL-23, as well as NF-κB.

Ligand	Protein	Interacting Residue *
Rutin	IL-17 receptor	Leu4 (π-alkyl), **Ser24** (HBA × 2), **Thr25** (HBA), **Asn91** (HBA), **Glu92** (HBA × 2), **Arg93** (HBD × 2), Leu 94 (CHBD), Leu94 (π-alkyl × 2), **Cys95** (HBD × 2), Cys95 (HBA)
	IL-22 receptor	**Tyr57** (HBA × 3), **Glu62** (HBA), Val83 (π-alkyl × 2), Val83 (π-lone Pair), **Glu90** (HBA), **Tyr92** (HBA), **Arg147** (HBA × 2)
	IL-23 receptor	**Ile154** (HBA), Thr152 (π-donor HBD × 2), Leu151 (π-alkyl), Thr152 (π-lone Pair), Tyr153 (π-π stacked) **Thr156** (HBA × 2), **Ser176** (HBA)
	NF-κB	**Gly50** (HBA × 2), **Ser222** (HBA), **Pro223** (HBA), **Ser226** (HBD), **Asn227** (HBD × 2), Lys229 (alkyl), Asp251 (π-anion), **Lys252** (HBD), Lys252 (π-σ)
Quercetin	IL-17 receptor	Ser168 (bump), **Gly169** (HBA × 2), **Trp172** (HBA), Cys259 (π-sulfur × 2), **Asp262** (HBA × 2), Cys263 (π-alkyl), Cys263 (π-sulfur), **Leu264** (HBD), Leu264 (π-σ), Leu264 (π-alkyl)
	IL-22 receptor	His27 (π-π stacked), **Lys29** (HBD), Lys29 (π-cation), Lys29 (π-σ), Lys29 (π-alkyl × 2), Lys108 (CHBD), Asp111 (π-anion), **Arg112** (HBA × 2)
	IL-23 receptor	**Glu137** (HBD), **Glu137** (HBA), **Ile219** (HBA), **Pro220** (HBA), **Ala223** (HBA), Trp307 (bump)
	NF-κB	**Leu228** (HBD), Lys229 (π-alkyl × 2), **Ile230** (HBD), **Ile230** (HBA), **Asp316** (HBA × 2), Val317 (π-lone Pair), Asp319 (π-anion × 2)

* Residues engaged in conventional hydrogen bonding are denoted in bold. HBD—hydrogen bond donor, HBA—hydrogen bond acceptor, π-donor HBD—π-donor hydrogen bond donor, CHBD—carbon–hydrogen bond donor, bump—unfavorable binding interaction.

**Table 5:** Focused molecular docking parameters for the best-docked binding poses of *G. verum* bioactive compounds.

Ligand–Protein Complex	ΔG_bind_ (kJ/mol)	K_i_ (M)	ΔG_Intermol. Energy_ _(vdw+Hbond+desolv)_ (kJ/mol)	ΔG_elec_ (kJ/mol)	ΔG_Final Intermol. Energy_ (kJ/mol)	ΔG_total_ (kJ/mol)	ΔG_tor_ (kJ/mol)	ΔG_unb_ (kJ/mol)
Rutin–MAPK2	−30.67	4.26 × 10^−6^	−47.40	−1.63	−49.04	−23.68	18.37	−23.68
Rutin–JAK2	−33.89	1.15 × 10^−6^	−49.62	−2.68	−52.26	−31.84	18.37	−31.84
Rutin–STAT3	−21.92	1.43 × 10^−4^	−37.66	−2.64	−40.29	−22.64	18.37	−22.64
Quercetin–MAPK2	−30.92	3.81 × 10^−6^	−36.61	−1.21	−37.82	−9.00	6.90	−9.00
Quercetin–JAK2	−29.25	7.57 × 10^−6^	−33.97	−2.13	−36.11	−6.95	6.90	−6.95
Quercetin–STAT3	−24.39	5.36 × 10^−5^	−30.38	−0.88	−31.25	−9.29	6.90	−9.29

**Table 6:** Overview of non-covalent binding interactions established by *G. verum* bioactive compounds and MAPK2, JAK2, as well as STAT3.

Ligand	Protein	Interacting Residue *
Rutin	MAPK2	Gly71 (π-σ), Gly73 (CHBD), Val78 (π-alkyl × 2), **Lys93** (HBD), Lys93 (π-alkyl), Met138 (π-sulfur), **Glu139** (HBA), Leu141 (CHBA), Glu190 (π-anion), Leu193 (π-alkyl), **Thr206** (HBD)
	JAK2	Leu855 (π-alkyl × 2), Val863 (π-alkyl × 2), Ala880 (π-alkyl), **Glu898** (HBA), Val911 (alkyl), Met929 (π-sulfur), Met929 (alkyl), **Glu930** (HBA), **Ser936** (HBD), **Ser936** (HBA), **Asp939** (HBA × 2), **Arg980** (HBA × 2), **Asn981** (HBA), Leu983 (π-σ × 2), Leu983 (alkyl), **Asp994** (HBD)
	STAT3	Val637 (π-σ), **Glu638** (HBA), Glu638 (CHBA × 2), Glu638 (π-anion × 2), Pro639 (CHBD), Pro639 (alkyl), **Gln644** (HBA), Tyr657 (π-π T-shaped)
Quercetin	MAPK2	**Ile74** (HBD), Val78 (π-alkyl × 2), Ala91 (π-alkyl), Met138 (π-alkyl), **Glu139** (HBA × 2), **Glu190** (HBA), Leu193 (π-alkyl), Thr206 (π-σ), **Asp207** (HBA)
	JAK2	Leu855 (π-σ), Leu855 (π-alkyl × 2), Val863 (π-alkyl), Ala880 (π-alkyl), Met929 (π-sulfur), **Glu930** (HBA), **Leu932** (HBD × 2), **Leu932** (HBA), **Asp939** (HBA × 2), Leu983 (π-σ), Leu983 (π-alkyl)
	STAT3	**Ser636** (HBA), Val637 (π-σ × 2), **Glu638** (HBD), Glu638 (π-anion), Glu638 (π-σ), Tyr640 (π-lone pair), **Gln644** (HBA × 2)

* Residues engaged in conventional hydrogen bonding are denoted in bold. HBD—hydrogen bond donor, HBA—hydrogen bond acceptor, CHBD—carbon–hydrogen bond donor, CHBA—carbon–hydrogen bond acceptor.

## Data Availability

Data is contained within the article and Supplementary Materials.

## Supplementary Materials

The following supporting information can be downloaded at: https://www.mdpi.com/article/10.3390/ijms26157290/s1.

## References

[1] Yamanaka K., Yamamoto O., Honda T. (2021). Pathophysiology of psoriasis: A review. J. Dermatol..

[2] Langley R.G., Krueger G.G., Griffiths C.E. (2005). Psoriasis: Epidemiology, clinical features, and quality of life. Ann. Rheum. Dis..

[3] Lu Y., Yang Y., Zhang J., Zhang H., Ma C., Tang X., Wu J., Li L., Wei J., Chen H. (2021). Anti-Angiogenic Efficacy of PSORI-CM02 and the Associated Mechanism in Psoriasis In Vitro and In Vivo. Front. Immunol..

[4] Corbic M., Jakovljevic V., Nikolic M., Jeremic N., Bradic J., Novakovic J., Kocovic A., Savic M., Tadic V., Zugic A. (2025). *Galium verum* L. extract mitigates cardiovascular events in psoriasis rats. Mol. Cell. Biochem..

[5] Dobrică E.C., Cozma M.A., Găman M.A., Voiculescu V.M., Găman A.M. (2022). The Involvement of Oxidative Stress in Psoriasis: A Systematic Review. Antioxidants.

[6] Shakoei S., Nakhjavani M., Mirmiranpoor H., Motlagh M.A., Azizpour A., Abedini R. (2021). The Serum Level of Oxidative Stress and Antioxidant Markers in Patients with Psoriasis: A Cross-sectional Study. J. Clin. Aesthet. Dermatol..

[7] Bilski R., Kupczyk D., Woźniak A. (2024). Oxidative Imbalance in Psoriasis with an Emphasis on Psoriatic Arthritis: Therapeutic Antioxidant Targets. Molecules.

[8] Armstrong A.W., Read C. (2020). Pathophysiology, Clinical Presentation, and Treatment of Psoriasis: A Review. JAMA.

[9] Bakshi H., Nagpal M., Singh M., Dhingra G.A., Aggarwal G. (2020). Treatment of Psoriasis: A Comprehensive Review of Entire Therapies. Curr. Drug Saf..

[10] Nowak-Perlak M., Szpadel K., Jabłońska I., Pizon M., Woźniak M. (2022). Promising Strategies in Plant-Derived Treatments of Psoriasis-Update of In Vitro, In Vivo, and Clinical Trials Studies. Molecules.

[11] Talbott W., Duffy N. (2015). Complementary and alternative medicine for psoriasis: What the dermatologist needs to know. Am. J. Clin. Dermatol..

[12] Yargholi A., Shirbeigi L., Rahimi R., Mansouri P., Ayati M.H. (2021). The effect of Melissa officinalis syrup on patients with mild to moderate psoriasis: A randomized, double-blind placebo-controlled clinical trial. BMC Res. Notes.

[13] Dabholkar N., Rapalli V.K., Singhvi G. (2021). Potential herbal constituents for psoriasis treatment as protective and effective therapy. Phytother. Res..

[14] Halim S.A., Khan A., Csuk R., Al-Rawahi A., Al-Harrasi A. (2020). Diterpenoids and Triterpenoids From Frankincense Are Excellent Anti-psoriatic Agents: An in silico Approach. Front. Chem..

[15] Alanzi A.R., Alsalhi M.S., Mothana R.A., Alqahtani J.H., Alqahtani M.J. (2024). Insilico discovery of novel Phosphodiesterase 4 (PDE4) inhibitors for the treatment of psoriasis: Insights from computer aided drug design approaches. PLoS ONE.

[16] Ibezim A., Onah E., Dim E.N., Ntie-Kang F. (2021). A computational multi-targeting approach for drug repositioning for psoriasis treatment. BMC Complement. Med. Ther..

[17] Bradic J., Jeremic N., Petkovic A., Jeremic J., Zivkovic V., Srejovic I., Sretenovic J., Matic S., Jakovljevic V., Tomovic M. (2020). Cardioprotective effects of *Galium verum* L. extract against myocardial ischemia-reperfusion injury. Arch. Physiol. Biochem..

[18] Al-Snafi A.E. (2018). *Galium verum*–A review. Indo Am. J. Pharm. Sci..

[19] Vuletic M., Jakovljevic V., Zivanovic S., Papic M., Papic M., Mladenovic R., Zivkovic V., Srejovic I., Jeremic J., Andjic M. (2022). The Evaluation of Healing Properties of *Galium verum*-Based Oral Gel in Aphthous Stomatitis in Rats. Molecules.

[20] Bradic J., Andjic M., Novakovic J., Kocovic A., Tomovic M., Petrovic A., Nikolic M., Mitrovic S., Jakovljevic V., Pecarski D. (2023). Lady’s Bedstraw as a Powerful Antioxidant for Attenuation of Doxorubicin-Induced Cardiotoxicity. Antioxidants.

[21] Farcas A.D., Mot A.C., Zagrean-Tuza C., Toma V., Cimpoiu C., Hosu A., Parvu M., Roman I., Silaghi-Dumitrescu R. (2018). Chemo-mapping and biochemical-modulatory and antioxidant/prooxidant effect of *Galium verum* extract during acute restraint and dark stress in female rats. PLoS ONE.

[22] Laanet P.-R., Saar-Reismaa P., Jõul P., Bragina O., Vaher M. (2023). Phytochemical Screening and Antioxidant Activity of Selected Estonian Galium Species. Molecules.

[23] Lakic N.S., Mimica-Dukic N.M., Isak J.M., Bozin B.N. (2010). Antioxidant properties of *Galium verum* L. (Rubiaceae) extracts. Centr. Eur. J. Biol..

[24] Turcov D., Barna A.S., Trifan A., Blaga A.C., Tanasă A.M., Suteu D. (2022). Antioxidants from *Galium verum* as Ingredients for the Design of New Dermatocosmetic Products. Plants.

[25] Chen H., Lu C., Liu H., Wang M., Zhao H., Yan Y., Han L. (2017). Quercetin ameliorates imiquimod-induced psoriasis-like skin inflammation in mice via the NF-κB pathway. Int. Immunopharmacol..

[26] Zhai M., Chen T., Shao M., Yang X., Qi Y., Kong S., Jiang L., Yang E. (2025). Unveiling the molecular mechanisms of Haitang-Xiaoyin Mixture in psoriasis treatment based on bioinformatics, network pharmacology, machine learning, and molecular docking verification. Comput. Biol. Chem..

[27] Sundarrajan S., Nandakumar M.P., Prabhu D., Jeyaraman J., Arumugam M. (2020). Conformational insights into the inhibitory mechanism of phyto-compounds against Src kinase family members implicated in psoriasis. J. Biomol. Struct. Dyn..

[28] Dhanabal S.P., Muruganantham N., Basavaraj K.H., Wadhwani A., Shamasundar N.M. (2012). Antipsoriatic activity of extracts and fractions obtained from Memecylon malabaricum leaves. J. Pharm. Pharmacol..

[29] Wu P., Liu Y., Zhai H., Wu X., Liu A. (2024). Rutin alleviates psoriasis-related inflammation in keratinocytes by regulating the JAK2/STAT3 signaling. Skin. Res. Technol..

[30] Forsyth T., Kearney P.C., Kim B.G., Johnson H.W., Aay N., Arcalas A., Brown D.S., Chan V., Chen J., Du H. (2012). SAR and in vivo evaluation of 4-aryl-2-aminoalkylpyrimidines as potent and selective Janus kinase 2 (JAK2) inhibitors. Bioorg. Med. Chem. Lett..

[31] Argiriadi M.A., Ericsson A.M., Harris C.M., Banach D.L., Borhani D.W., Calderwood D.J., Demers M.D., Dimauro J., Dixon R.W., Hardman J. (2010). 2,4-Diaminopyrimidine MK2 inhibitors. Part I: Observation of an unexpected inhibitor binding mode. Bioorg. Med. Chem. Lett..

[32] Bai L., Zhou H., Xu R., Zhao Y., Chinnaswamy K., McEachern D., Chen J., Yang C.Y., Liu Z., Wang M. (2019). A Potent and Selective Small-Molecule Degrader of STAT3 Achieves Complete Tumor Regression In Vivo. Cancer Cell.

[33] Xu F., Xu J., Xiong X., Deng Y. (2019). Salidroside inhibits MAPK, NF-κB, and STAT3 pathways in psoriasis-associated oxidative stress via SIRT1 activation. Redox Rep..

[34] Liu A., Zhao W., Zhang B., Tu Y., Wang Q., Li J. (2020). Cimifugin ameliorates imiquimod-induced psoriasis by inhibiting oxidative stress and inflammation via NF-κB/MAPK pathway. Biosci. Rep..

[35] Lin J., Fang Y., Cao Y., Ma L., Tao M., Wang X., Li Y., Qing L. (2023). Zerumbone attenuates the excessive proliferation of keratinocytes in psoriasis mice through regulating NLRP3/NF-κB pathway. Toxicol. Res..

[36] Liu A., Zhang B., Zhao W., Tu Y., Wang Q., Li J. (2021). Catalpol ameliorates psoriasis-like phenotypes via SIRT1 mediated suppression of NF-κB and MAPKs signaling pathways. Bioengineered.

[37] Pleńkowska J., Gabig-Cimińska M., Mozolewski P. (2020). Oxidative Stress as an Important Contributor to the Pathogenesis of Psoriasis. Int. J. Mol. Sci..

[38] Blagov A., Sukhorukov V., Guo S., Zhang D., Eremin I., Orekhov A. (2023). The Role of Oxidative Stress in the Induction and Development of Psoriasis. Front. Biosci. (Landmark Ed.).

[39] Bijelić K., Srdjenović Čonić B., Prpa B., Pilija V., Vukmirović S., Kladar N. (2024). The Potential of Hemp Extracts to Modify the Course of Oxidative-Stress Related Conditions. Plants.

[40] Bobo-García G., Davidov-Pardo G., Arroqui C., Vírseda P., Marín-Arroyo M.R., Navarro M. (2015). Intra-laboratory validation of microplate methods for total phenolic content and antioxidant activity on polyphenolic extracts, and comparison with conventional spectrophotometric methods. J. Sci. Food Agric..

[41] Acharya K. (2017). Simplified methods for microtiter based analysis of in vitro antioxidant activity. Asian J. Pharm..

[42] Ramírez-García O., Salinas-Moreno Y., Santillán-Fernández A., Sumaya-Martínez M.T. (2022). Screening antioxidant capacity of Mexican maize (Zea mays L.) landraces with colored grain using ABTS, DPPH and FRAP methods. Cereal Res. Commun..

[43] Johnson J.B., Mani J.S., Naiker M. (2023). Microplate methods for measuring phenolic content and antioxidant capacity in chickpea: Impact of shaking. Eng. Proc..

[44] Bolanos de la Torre A.A., Henderson T., Nigam P.S., Owusu-Apenten R.K. (2015). A universally calibrated microplate ferric reducing antioxidant power (FRAP) assay for foods and applications to Manuka honey. Food Chem..

[45] Morris G.M., Ruth H., Lindstrom W., Sanner M.F., Belew R.K., Goodsell D.S., Olson A.J. (2009). AutoDock4 and AutoDockTools4: Automated docking with selective receptor flexibility. J. Comput. Chem..

[46] Schrödinger L., DeLano W. PyMOL. https://www.pymol.org/pymol.

[47] Zhan Y.P., Chen B.S. (2023). Drug Target Identification and Drug Repurposing in Psoriasis through Systems Biology Approach, DNN-Based DTI Model and Genome-Wide Microarray Data. Int. J. Mol. Sci..

[48] Potestio L., Tommasino N., Lauletta G., Martora F., Megna M. (2024). Psoriasis and Molecular Target Therapies: Evidence of Efficacy in Preventing Cardiovascular Comorbidities. Dermatol. Ther..

[49] Liu S., Dakin L.A., Xing L., Withka J.M., Sahasrabudhe P.V., Li W., Banker M.E., Balbo P., Shanker S., Chrunyk B.A. (2016). Binding site elucidation and structure guided design of macrocyclic IL-17A antagonists. Sci. Rep..

[50] Bleicher L., de Moura P.R., Watanabe L., Colau D., Dumoutier L., Renauld J.C., Polikarpov I. (2008). Crystal structure of the IL-22/IL-22R1 complex and its implications for the IL-22 signaling mechanism. FEBS Lett..

[51] Bloch Y., Bouchareychas L., Merceron R., Składanowska K., Van den Bossche L., Detry S., Govindarajan S., Elewaut D., Haerynck F., Dullaers M. (2018). Structural Activation of Pro-inflammatory Human Cytokine IL-23 by Cognate IL-23 Receptor Enables Recruitment of the Shared Receptor IL-12Rβ1. Immunity.

[52] Cramer P., Larson C.J., Verdine G.L., Müller C.W. (1997). Structure of the human NF-kappaB p52 homodimer-DNA complex at 2.1 A resolution. EMBO J..

[53] Biovia D.S., Berman H.M., Westbrook J., Feng Z., Gilliland G., Bhat T.N. (2000). Dassault Systèmes BIOVIA, Discovery Studio Visualizer, V. 17.2.

[54] Milevic A., Simic M., Tomovic M., Rankovic M., Jakovljevic V., Bradic J. (2022). The effects of methanol extract of *Galium verum* L on cardiac redox state in hypertensive rats. Braz. J. Pharm. Sci..

[55] Sretenovic J., Joksimovic Jovic J., Srejovic I., Zivkovic V., Mihajlovic K., Labudovic-Borovic M., Trifunovic S., Milosevic V., Lazic D., Bolevich S. (2021). Morphometric analysis and redox state of the testicles in nandrolone decanoate and swimming treated adult male rats. Basic. Clin. Androl..

[56] Ohkawa H., Ohishi N., Yagi K. (1979). Assay for lipid peroxides in animal tissues by thiobarbituric acid reaction. Anal. Biochem..

[57] Green L.C., Wagner D.A., Glogowski J., Skipper P.L., Wishnok J.S., Tannenbaum S.R. (1982). Analysis of nitrate, nitrite and [15 N] nitrate in biological fluids. Anal. Biochem..

