# Supplementary material for: The Therapeutic Potential of Galium verum for Psoriasis: A Combined Phytochemical, In Silico, and Experimental Approach
Source: Int J Mol Sci. 2025 Jul 28;26(15):7290. doi: 10.3390/ijms26157290 (PMC12347447; doi:10.3390/ijms26157290)
Supplement: Supplementary file 1 [file ijms-26-07290-s001.zip › ijms-3760717-supplementary.pdf]

Supplementary Information

The Therapeutic Potential of *Galium verum* for Psoriasis: A Combined Phytochemical, In Silico, and Experimental Approach

Branislava Daskalovic [1], Vladimir Jakovljevic [2,3,4], Sergej Bolevic [4], Marijana Andjic [3,5], Jovana Bradic [3,5], Aleksandar Kocovic [3,5], Milos Nikolic [5], Nikola Nedeljkovic [5], Jovan Milosavljevic [2], Jovan Baljak [6], Milos Krivokapic [7], Svetlana Trifunovic [8] and Jasmina Sretenovic [2,3,*]

[1] Goodwill Pharma d.o.o., 24000 Subotica, Serbia
[2] Department of Physiology, Faculty of Medical Sciences, University of Kragujevac, Svetozara Markovica 69, 34000 Kragujevac, Serbia
[3] Center of Excellence for Redox Balance Research in Cardiovascular and Metabolic Disorders, 34000 Kragujevac, Serbia
[4] Department of Human Pathology, 1st Moscow State Medical University IM Sechenov, Trubetskaya Str. 2, 119992 Moscow, Russia
[5] Department of Pharmacy, Faculty of Medical Sciences, University of Kragujevac, Svetozara Markovica 69, 34000 Kragujevac, Serbia
[6] Department of Pharmacy, Faculty of Medicine, University of Novi Sad, Hajduk Veljkova 3, 21000 Novi Sad, Serbia
[7] Faculty of Medicine, University of Montenegro, Kruševac bb, 81000 Podgorica, Montenegro
[8] Department of Cytology, Institute for Biological Research "Sinisa Stankovic"—National Institute of Republic of Serbia, University of Belgrade, Bulevar Despota Stefana 142, 11000 Belgrade, Serbia
* Correspondence: drj.sretenovic@gmail.com

Contents Macroscopic and microscopic images of psoriatic skin changes in rats

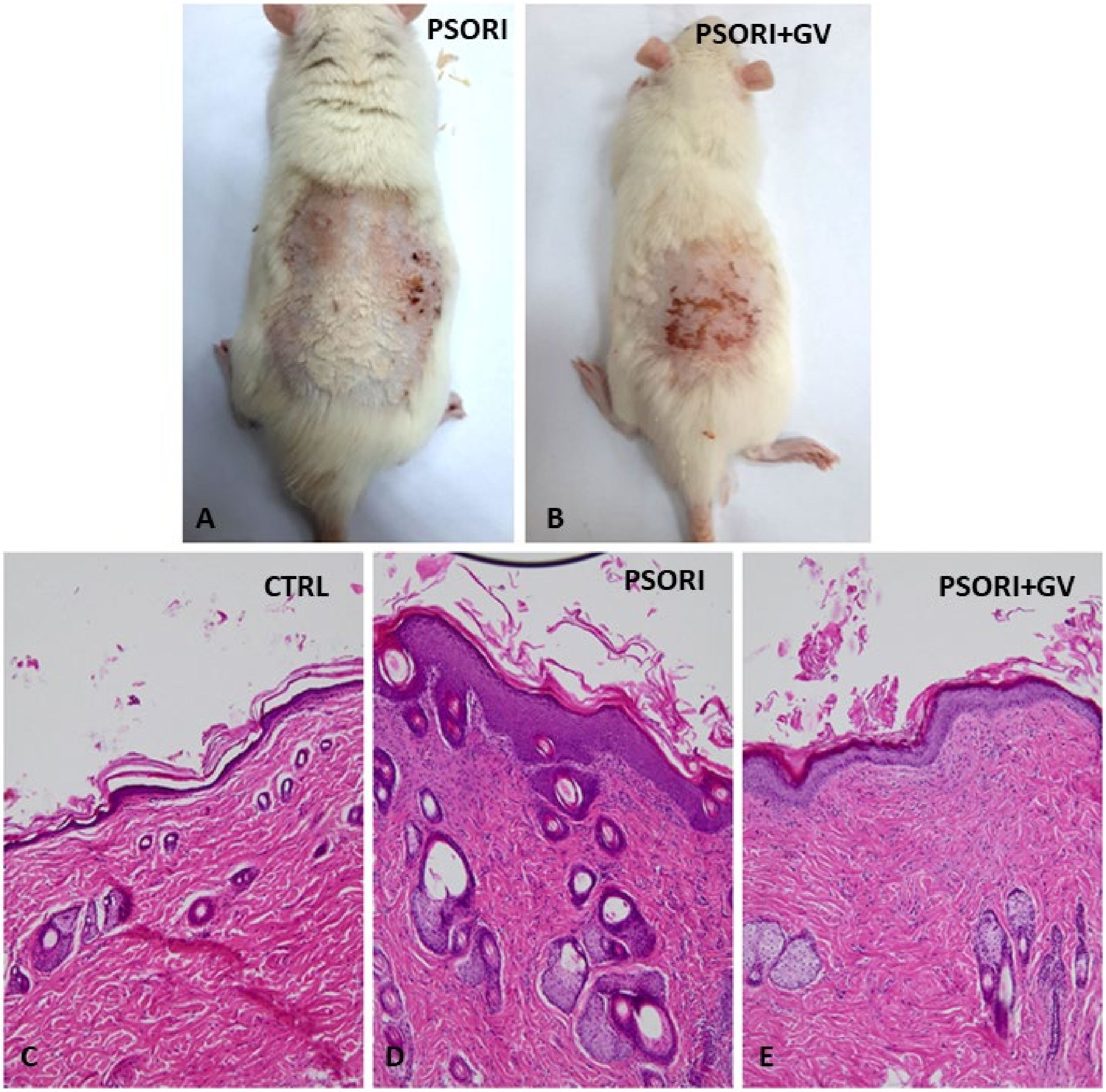

Figure S1. Macroscopic (first row) and microscopic (second row) images of psoriatic skin changes in rats: rat skin after psoriasis induction (A); rat skin after 7 days of treatment with G. verum extract (B); control group skin, H&E staining (C); psoriasis group skin, H&E staining (D); G. verum-treated group skin, H&E staining (E); control group skin, (Magnigication 100×).
